# An ecological study of regional variation in work injuries among young workers

**DOI:** 10.1186/1471-2458-7-91

**Published:** 2007-05-23

**Authors:** F Curtis Breslin, Peter Smith, James R Dunn

**Affiliations:** 1 Institute for Work & Health, 481 University Ave., Suite 800, Toronto, Ontario, Canada; 2 Dept of Public Health, University of Toronto, Ontario, Canada; 3 Institute of Medical Science, University of Toronto, Ontario, Canada; 4 Research on Inner City Health, St. Michael's Hospital, Toronto, Ontario, Canada; 5 Dept of Geography, University of Toronto, Ontario, Canada

## Abstract

**Background:**

The investigation of geographic variation in occupational injuries has received little attention. Young workers 15 to 24 years are of particular concern because they consistently show elevated occupational injury rates compared to older workers. The present study sought to: (a) to describe the geographic variation of work injuries; (b) to determine whether geographic variation remained after controlling for relevant demographic and job characteristics; (c) to identify the region-level factors that correlate with the geographic variation.

**Methods:**

Using workers compensation claims and census data, we estimated claim rates per 100 full-time equivalents for 15 to 24 year olds in 46 regions in Ontario. A total of 21 region-level indicators were derived primarily from Census and Labour Force Survey data to reflect social and material deprivation of the region as well as demographic and employment characteristics of youth living in those areas.

**Results:**

Descriptive findings showed substantial geographic variation in young worker injury rates, even after controlling for several job and demographic variables. Region-level characteristics such as greater residential stability were associated with low work injury rates. Also, regions with the lowest claim rates tended to have proportionally fewer cuts and burns than high-claim-rate regions.

**Conclusion:**

The finding of substantial geographic variation in youth claim rates even after controlling for demographic and job factors can aid in targeting prevention resource. The association between region-level indicators such as residential stability and youth work injury suggests that work injury prevention strategies can be integrated with other local economic development measures. The findings partially support the notion that work safety measures may be unevenly distributed with respect to regional socio-economic factors.

## Background

There is a growing body of evidence suggesting that many youth and adult health outcomes are not merely the result of the differential distribution of individual-level risk factors, but are also attributable to aspects of the social and economic environment (i.e., context) [1-7]. Some studies have shown modest associations (beyond the influence of individual-level factors) between area-level socioeconomic factors and health outcomes such as general health and hypertension (for a review see [8]). Other health outcomes such as coronary heart disease have shown marked variation due to area-level socioeconomic characteristics [1,9].

Despite this proliferation of interest in contextual effects on health generally, investigation of contextual effects through geographic variation in occupational injuries has received little attention. The purpose of the present study was to characterize geographic variation in work injury rates among young workers, and to assess whether area-level social and economic factors are associated with geographic variation in occupational injuries.

The importance of this kind of analysis is threefold: 1) characterizing the geographic pattern of youth injuries allows for the geographical targeting of resources and interventions to reduce this preventable cause of morbidity and mortality; 2) although a straightforward ecological analysis, the results of this study will be useful in informing subsequent studies with data that allow for more powerful inferences; and 3) although most texts on epidemiologic methods denigrate ecological studies because of their vulnerability to the ecological fallacy, a number of scholars have successfully argued that there are merits to this method. Notably, a number of authors have raised the importance of a 'higher level' of analysis and effects on health, namely the 'social facts' [10,11] operating at the level of 'whole populations' [12,13]. This perspective implies that there are population or social risks that cannot be inferred from individual data; indeed, the ecological study is a good methodology for detecting the possibility of such effects [3,14,15].

### Background on young workers

Many young Canadians are engaged in paid employment. Employment rates from the Labour Force Survey show that 44.7% of Canadian adolescents (15 to 19 years old) and 70.9% of young adults (20 to 24 years olds) were employed in any given month during 2004 [16].

Young workers occupy a particular niche in the Canadian labour market that is characterized by part-time, temporary work, and concentration in certain jobs and industries. Canadian adolescents and young adults work an average of 21.2 and 32.4 hours per week, respectively [16]. Even during the school year (September to May), 41.7% of Canadian adolescents and 29.2% of young adults reported having worked in their current job for six months or less [17].

Canadian adolescents are most likely to hold sales and service jobs such as food and beverage, cashier, and retail sales (67%) and clerical/administration jobs (8.5%). Nevertheless, there is some representation in more hazardous jobs such as trades/transportation (e.g., construction; 7.7%) and primary industry (e.g., agriculture; 7.8%) [17]. Young adults are less concentrated in sales and service jobs (37.6%), and more likely to work in clerical/administration (12.8%), trades/transportation (12.8%) and primary industry (9.6%) jobs than adolescents. These similarities among adolescent and young adult workers are partly due to the fact that the school to work transition is extending into the mid to late 20's [18]. Consequently, both age groups provide valuable information about the initial stages of work life.

Work injuries and illnesses among young people 15 to 24 years old entering the formal labour market are a public health concern. In developed countries, both teenagers and young adults have rates of work injury that are typically 1.2 to two times that of older workers [19-21]. This elevated risk is more marked for young males than young females. Because young workers are more likely to work part-time or seasonally, injury rates based on number of hours worked (rather than per worker) show an elevated youth risk more consistently [22,23].

Many of the work injuries youth sustain have a clear health and economic consequence. Fifteen to 26% of injured adolescent workers surveyed reported permanent impairments such as chronic pain, scarring, sensory loss, and loss of range of motion. [24,25]. Sixteen to 24 year olds who sustained a work injury showed significantly lower earnings in the year after the injury than their uninjured counterparts [26].

Among young workers, individual characteristics are associated with claim rates. For example, young males sustain work injuries at about twice the rate of young females [27-34]. In seven studies examining adolescent and young adult claim rates by industry, the retail/wholesale, manufacturing, agriculture, and construction industrial sectors typically had higher claim rates than the service sector [27-34]. With regard to occupational groups, young workers in manual jobs such as handlers/laborers, janitors/cleaners and food service exhibited higher work injury rates than youth in sales or administrative/clerical jobs [27,29,30]. Previous research has shown that Ontario young workers have a similar set of demographic and work-related factors associated with their risk of work injury as found in young worker studies elsewhere in North America [34].

Geographic variation in youth work injury rates, and the factors underlying these variations, are just beginning to be explored. A Canada-wide survey showed that even when demographic and work factors were statistically controlled, 15 to 24 year olds in a western province were twice as likely to be injured at work compared to youth in Ontario, a large eastern province [35]. Brooks and Davis [36] conducted an analysis of Massachusetts workers' compensation claims for teenagers in 45 regions in the state. The highest claim rates were found in the southeast region of the state. Compositional differences in jobs were suggested because hazardous industries such as apparel manufacturing are concentrated in this region. Also suggesting potential regional differences in deprivation, the southeast region of the state had a lower per capita income and higher unemployment rate than the state as a whole. However, there was no attempt in this study to control for compositional differences and formally assess the association between region-level indicators and variation in adjusted claim rates.

### Rationale for level of aggregation

One conceptual and methodological issue for geographic studies is the level of aggregation of areas [3]. The census division, the level of aggregation for the present study, is a "general term applying to areas established by provincial law, which are intermediate geographical areas between the municipality (census subdivision) and the province. Usually they are created to facilitate regional planning and the provision of services which can be more effectively delivered on a scale larger than a municipality" [37].

Examining regional variation at this subprovincial level is appropriate for occupational health data as labour markets are significantly shaped by geography [38]. Aggregating to this level makes it possible to capture a mix of industries within the local regional economy, as compared to a detailed neighbourhood analysis where that diversity would be attenuated. Moreover, census divisions (CDs) will, on average, approximate regional labour markets, although in the largest urban areas the labour market may consist of several CDs because of the size of the city and density of employers. Regional measures of the labour market and socioeconomic status also offer the potential advantage of greater stability than neighbourhood- or workplace-level indicators [39].

### Indicators and pathways for geographic variation

Regional variation in work injury rates can be due to both compositional and contextual factors. Variation can occur because regions may have demographically different subgroups of young workers or young workers hold different types of jobs. For example, the economy in more rural regions of the country lead young workers to have proportionally more jobs in the agricultural sector than in urban areas [17].

Geographic variation can also occur due to contextual factors. Contextual factors refer to the physical, social or economic aspects of a workplace or region (.([40]. The development of indices to summarize the socio-economic status of whole regions is a significant scholarly enterprise, with indices such at the Carstairs index, the Townsend deprivation score and others now widely used [41]. For the purposes of this study, we adopted the material and social deprivation indices developed in Quebec. According to Pampalon and Raymond [42], two forms of area deprivation, material and social, are significant for health at the regional level and we hypothesize that these same factors could influence work injury rates. Material deprivation involves the lack of economic resources to provide or access goods and services relevant to health, and is reflected in level of education and income indices. Relative material deprivation in a region could limit the capability of local businesses to invest in safer equipment and to set up the infrastructure to effectively disseminate work safety information.

Social deprivation refers to the quality of relationships in the workplace and community [42]. Common indicators of this construct are the transience of residents in the area and family structure (e.g., single parent families). With regard to workplaces, relevant aspects of the social environment include workplace size and the degree of unionization [43,44], which reflects the capability for collective action. Relative social deprivation can exert influence on the workplace and health through a weak safety culture and lack of adherence to safety practices. Less socially cohesive areas may also lead to higher manager/employee turnover and poorer safety education and training.

The main analytic focus of the research was on geographic variation of youth work injuries and the relationship between that variation and area social/economic deprivation. Substantial geographic variation even after controlling for compositional differences raises the possibility of the presence of contextual factors. Using compensation claims data of 15 to 24 year old workers from a Canadian province, the present study had three objectives: (a) to describe the geographic variation of work injuries; (b) to determine whether geographic variation remained after controlling for relevant demographic and job characteristics; (c) to identify the region-level factors that correlate with the geographic variation.

## Methods

This study was approved by the University of Toronto Health Sciences Ethics Committee (#14978). Data for this project was taken from numerous sources including: administrative data from the Ontario Workplace Safety & Insurance Board; custom tabulations from the 2000 Labour Force Survey; the 2001 Canadian Census; the Survey of Labour and Income Dynamics (SLID); and the Education, Quality and Accountability Office of Ontario.

### Data Sources

The Canadian Census is conducted every 5-years by Statistics Canada. The Census contains both short form and long form questionnaires. The short form contains seven questions: the respondent's name, sex, age, marital and common-law status, family and household relationships, and mother tongue. The long form includes the seven questions from the short questionnaire plus 52 additional questions. These include questions on the average number of weeks worked in the previous year, type of occupation and industry each respondent works in, information on family composition, socio-demographic composition, education, income and geographic mobility in the last year and over the last five years. Four in five Canadian households receive the short form, with the other 20% receiving the long form.

The Labour Force Survey follows a complex, rotating panel sample design to efficiently estimate monthly changes in the Canadian labour force [45]. The sample size covered by the Labour Force Survey is approximately 54,000 households each month. Each household is surveyed monthly, for six months. The Labour Force Survey contains information on industry, occupation, and hours of work in the previous week

Begun in 1993, the SLID is a longitudinal survey of representative samples of Canadian households. The survey consists of a series of six-year panels, with a new panel being introduced every three years to replace the oldest panel. Each panel contains approximately 15,000 households with about 31,000 respondents aged 16 years old and over. The population for each SLID panel is recruited from participants to the Labour Force Survey.

The Education, Quality and Accountability Office website published the results of the Ontario Secondary School Literacy test (OSSLT). The OSSLT assesses the reading and writing skills that students are expected to have acquired, according to the Ontario school curriculum, by the end of grade nine. The OSSLT is administered to students within all schools in Ontario.

## Main Outcome

### Estimates for lost-time injury claims

Lost-time injury claims information for 15 to 24 year olds that occurred in 2000 was gathered from the Ontario Workplace Safety and Insurance Board (WSIB). The present study combined teenage and young adult claims because: a) both age groups are in the school to work transition (as noted above); b) previous research has shown comparable work injury rates for both age groups [21,34,46].; and c) the precision and stability of the claim rate estimates by region was aided by using both age groups.

The WSIB is the sole provider of workers compensation in Ontario and covers approximately 65% – 70% of Ontario labour force participants [47]. The remaining 30 to 35% not covered included those self-employed, domestic workers, federal government workers, the finance industry, and workers associated with inter-provincial commerce.

All WSIB covered workers in Ontario are required to submit lost-time claims for any workplace injury that requires health care and/or (1) results in an absence from their regular work, (2) requires modified duties at less than regular pay, (3) requires modified duties at regular pay for more than seven calendar days after their accident, (4) results in earning less than regular pay at regular work. Injuries requiring only first aid, such as cleaning minor cuts, scrapes or scratches, applying bandages or dressings, or applying splints, do not require submission of a lost-time claim.

Along with socio-demographic information such as age and gender, and workplace related information such as industrial sector, each lost-time claim submitted to the WSIB contains information on the census division in which the claimant lived and the location where the injury occurred. Injuries were classified into census divisions first using the living location of each claimant; if this information was not available the location where the injury occurred was used (1.4% of claims).

### Estimates of Full-time-equivalents worked in 2000

Estimates of the average number of weeks worked, stratified by census division, gender, full-time or part-time status, industry (goods and services) and occupation (manual, mixed and non-manual) were obtained for all 15 – 24 year olds in Ontario, working in industries with mandatory coverage from the Ontario WSIB (for further details on exclusions to WSIB coverage see [48]. For details on the occupational categories see [49]. Unfortunately, the Census only contains information on the hours of work in the previous week (in 2001). As this number of hours may not be applicable to each respondent's working hours in the previous year, similar data on the average number of hours worked per week for all 15 – 24 year olds in Ontario was obtained from the Labour Force Survey, by level of economic region (of which there are 11 in Ontario). These estimates were applied to all census divisions within a given economic region to estimate the number of full-time equivalents (FTEs), gender, occupation and industry within each census division.

We adjusted the claim rate for each census by occupation and gender using direct standardization [50]. That is, injury rates presented have taken into account differences in the gender and occupational composition of the labour force between census divisions. We decided not to further adjust for industrial composition, given the percent of the labour force working in goods-producing industries was highly correlated with the percent of the labour force working in manual occupations.

The map of regional claim rates was constructed using MapInfo. The grouping of regions by claim rate represented in the maps was derived using a software algorithm that computes the natural breaks in the distribution of the variable.

With regard to the types of injuries, the Canadian Work Injury Standard codes were used to classify the nature of the injury [51] into the following groups: cut, burns, fractures, amputations, sprains and strains, contusions, and other (e.g., concussions, exposures, multiples injuries).

## Independent variables

The indicators of material and social deprivation described below refer to the socio-economic conditions of the region. In addition, we are using the area-level indicators to examine demographic and employment characteristics of youth living in those areas.

A total of 43 independent variables were gathered from the Census through custom tabulations. Additional information for each region on the percent of the unionized labour force and the percent of the labour force working in small workplaces (for both 15 – 24 yr olds and 25+ yr olds) were gathered from the SLID [52]. Because of sample size limitations in low population density regions, we combined two cross sectional SLID files from 1998 and 2002 to estimate the number of workers employed in small workplaces (less than 20 employees), and the number of workers who were members of a union. Although the combined SLID panels contained a large number of employees (15 – 24 yr old N = 5,334, and total sample N = 35,302), we still had to combine three census divisions. These were Lennox and Addington County (CD 3511) which was combined with Prince Edward Division (CD 3513); Manitoulin district (CD 3546) which was combined with Sudbury district (CD 3549); and Haliburton district (CD 3551), which was combined with the district of Parry Sound (3552).

School performance data was estimated using the percent of students who passed the Ontario Secondary School Literacy Test. These data were obtained for each school board from the Ontario Education Quality and Accountability Office's web site. We conceptualized these data as an indicator of social resources in the area, a factor that could influence the type or quality of job that a young person might hold. A number of school boards crossed census divisions. Therefore, a list of all schools and their postal codes was obtained, and the pass mark for each school was imputed from the pass mark of the school board to which they belonged. The pass percentage for a given census was then allocated using the combined average pass mark from the schools within that census division.

## Analytic strategy

### Reduction of predictors

The original data set contained 47 different variables at the Census Division level. An initial analytic step was to examine the correlations between each of these variables. Items with high correlations (> 0.85) with other items were removed due to probable redundancy. The revised data set contained 21 variables at the census division level:

1. Population density (Census)

2. Percent of total labour force with coverage by a union or collective agreement (SLID)

3. Percent of total labour force working in workplaces with less than 20 employees (SLID)

4. Percent of 15 – 24 year old labour force working in workplaces with less than 20 employees (SLID)

5. Average labour market earnings (Census)

6. Percent of 15 – 24 year olds not currently studying with less than high school education (Census)

7. Percent of 25+ year olds not currently studying with less than high school education (Census)

8. Unemployment rate for 15 – 24 year olds (Census)

9. Percent of tenant households who spend 30% or more on rent (Census)

10. Percent of owner households who spend 30% or more on household expenses (Census)

11. Percent of school children who successfully completed the Ontario Secondary School Literacy Test (OSSLT) in 2000–01 (Education, Quality and Accountability Office website)

12. Percent of families with a single mother and children under the age of 14 years (Census)

13. Percent of private dwellings that are owned (Census)

14. Average value of dwellings (Census)

15. Ratio of population aged over 65 years to the population aged 15 – 64 years (Census)

16. Percent of population who have lived at the same address for the last five years (Census)

17. Percent of the population who have lived at the same address for the last year (Census)

18. Percent of population who lived outside of Canada five years ago (Census)

19. Population growth between 1996 and 2001 (Census)

20. Percent of population with only French language ability (Census)

21. Percent of population with Aboriginal identity (Census)

The variables for population density, French only language ability and aboriginal identity were skewed and kurtotic. Therefore they were each log transformed before correlation analysis and factor analysis were undertaken.

We first examined if census divisions could be characterized by combinations of indicators of deprivation and labour market variables. The initial exploratory factor analysis revealed five factors. The percent of population with French only language was dropped as it loaded, by itself, onto only one factor. In a subsequent model the percentage of tenant households who spend more than 30% on rent only loaded onto one factor, by itself, and was subsequently removed as well. Our final factor analysis revealed four factors.

We then created factor scores for each of the four factors by taking only those variables with a loading of greater than 0.5 on a given factor. In two cases variables did not have loadings of over 0.50 with any factor. In these situations (for union membership and education for 25+ yrs) these variables were included in the factor that they loaded most strongly on. In two further cases (the percentage of household spending 30% or more of household income on expenses and the percent of dwellings that were owned) variables loaded onto two factors. We then performed four additional factor analyses, taking only the variables assigned to a given factor and constraining the number of factors to one, to produce factor scores, using PROC FACTOR in SAS version 9.1.

### Regression analyses

We then performed two regression analysis; the first examined the relationship between each of the factors and the adjusted injury rate within a census division; and the second examined the relationship between each of the variables contained within a given factor and the adjusted injury rate separately, with a final model including only the variables that were significantly associated with the adjusted injury rate. Because of the different size of each census divisions all regression and correlation analyses were weighted, using a relative weight based on the size of the 15 – 24 year old labour force within a census division.

Examination of influence statistics demonstrated the larger labour markets of Peel (10% of total labour force), Toronto (17%) and York (6%) regions were influential over the correlations obtained, as assessed by Cooks D statistics. While we could remove such influential observations, given we are examining how census division level variables influence work-related lost-time claim rates, and the largest number of workers are in these divisions, we decided to keep them in our analyses, as conditions within these divisions would be expected to be influential on overall work-injury rates. Note that removal of these census divisions, while changing correlation estimates, only changed the significant relationships reported to non-significant relationships, and vice versa in three situations (single mother families would be significant at p < 0.10, value of dwellings would be significant at p < 0.05 and ratio of population aged over 65 to population aged 15 – 64 years would reduce in significance to p < 0.10).

## Results

In 2001, from workplaces with mandatory coverage, there were 13,744 accepted lost-time claims from 15 to 24 year olds in Ontario. Young males (n= 10,028) had more than double the number of claims as young females (n = 3716). Overall, the annual claim rate for this age group was 3.9 (95% confidence interval (CI) = 3.84, 3.97) per 100 FTEs.

Table 1 presents descriptive statistics for each of the independent variables included in our analyses, as well as the weighted correlation between each variable and the claim rate, adjusted for gender and occupational differences, across each census division. One key observation that comes from an examination of the table is the substantial range in the values of each variable across regions. For example, estimated rates of unemployment for young people are from 9 to 25%; values of dwelling vary by more than three-fold (87,314 to 298,018).

**Table 1:** Descriptive information on study variables and correlations between each and the adjusted injury rate within a census division

**Variable**	**Mean**	**Range**	Correlation with Adjusted* claim rate
Population density (log)	3.45	-1.6 – 8.3	**0.31†**
15 – 24 yr olds with only high school education	0.44	0.32 – 0.64	**-0.34†**
Households spending 30%+ on expenses	0.15	0.10 – 0.22	0.20
Dwelling ownership	0.74	0.51 – 0.86	-0.02
Same address – 5 yrs	0.61	0.52 – 0.70	**-0.49†**
Same address – 1 yr	0.87	0.83 – 0.90	-0.28‡
Outside Canada – 5 yrs	0.02	0.00 – 0.11	0.19
Percent union	0.27	0.08 – 0.45	-0.10
Unemployment 15 – 24 yrs	0.14	0.09 – 0.25	-0.22
Percent completing literacy test	0.59	0.43 – 0.70	0.15
Single mother families	0.06	0.04 – 0.08	0.10
Percent aboriginal (log)	-3.94	-5.65 – -1.05	-0.24
Average earnings	31,067	23,934 – 45,835	0.28‡
25+ years with only high school education	0.32	0.18 – 0.41	**-0.33†**
Value of dwellings	154,576	87,314 – 298,018	0.18
Ratio of pop 65+ to pop 25 – 64 years	0.22	0.11 – 0.32	**-0.47†**
Population growth 96 – 01	0.03	-0.09 – 0.23	**0.35†**
Percent of labour force in small workplaces	0.48	0.36 – 0.65	**-0.50†**
Percent of youth in small workplaces	0.52	0.32 – 0.88	**-0.31†**

* Adjusted for gender and occupation

†p <0.05 ‡

p < 0.10

Population density and population growth were both positively associated with the adjusted work injury rate. The percent of 15 – 24 year olds with high school education (not currently studying), the percent of the population who had lived at the same address for the last five years, the percent of adults with high school education, and both the percent of adults and 15 – 24 year olds working in small workplaces were all negatively associated with the adjusted injury rate.

**Figure 1:**
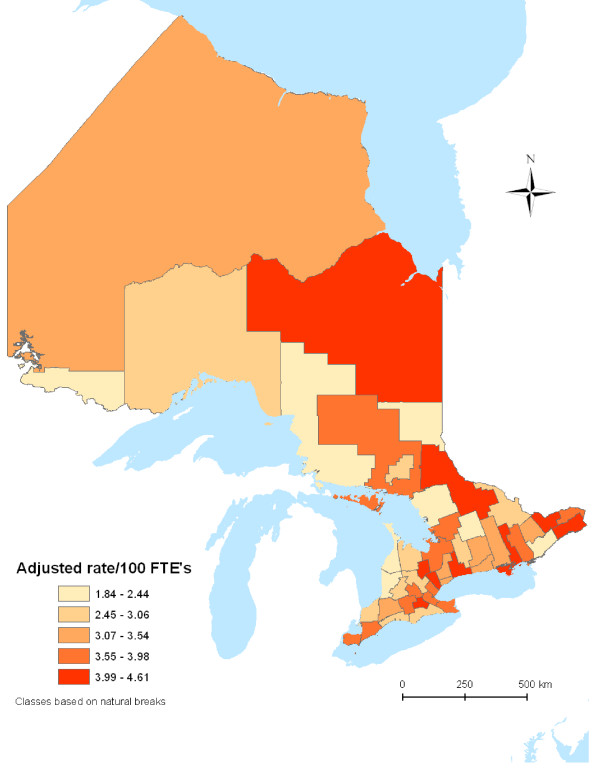
Work injury rates per 100 Full-time equivalents by census division among 15 to 24 year old workers adjusted for gender and occupation.

Figure 1 shows that the rates of youth work injury adjusted by gender and occupation still vary substantially from one region to another. Areas with the lowest injury rates were Rainy River District and Huron County (1.98 [CI= 1.2, 3.3] and 1.84 [CI= 1.5, 2.3] per 100 FTEs, respectively), while Cochrane District, Lennon & Addington County/Prince Edward Division and Dufferin Country had the highest rates (4.37 [CI = 3.6, 5.3], 4.41 [CI = 3.7, 5.3], and 4.61 [CI = 3.8, 5.6] per 100 FTEs, respectively). The values used to generate the map can be found in Appendix A [see additional file 1]. To link the names of divisions to areas of the maps, a legend is provided in Appendix B [see additional file 2].

Table 2 presents the factors and loadings produced through the factor analysis of predictors. Factor One was interpreted as characterizing low population density and deprivation features such as low youth education, low spending on household expenses and low population mobility. Factor Two appears to reflect primarily social and economic deprivation, with high youth unemployment, low household ownership, low literacy test completion, high single mother families, and high aboriginal population. However, high union membership was also part of this factor, which is not typically considered an indicator of deprivation. Factor Three was interpreted as a lack of deprivation cluster characterized by high earnings, high adult education, high value of dwellings, young population, and high population growth. Finally, Factor Four we labelled summer tourist areas because the areas loading highest on this factor are well known places for summer vacation (informally known as "cottage country"; see Table 3). A distinctive characteristic of these areas are the high percentage of small workplaces (see Table 2).

**Table 2:** Standardized regression coefficients for each factor*

**Variable**	**Factor One**	**Factor Two**	**Factor Three**	**Factor Four**
Population density (log)	-0.71			
15 – 24 yr olds with only high school education	0.84			
Households spending 30%+ on expenses	-0.61			0.66
Dwelling ownership	0.65	-0.71		
Same address – 5 yrs	0.58			
Same address – 1 yr	0.66			
Outside Canada – 5 yrs	-0.70			
Percent union		0.48		
Unemployment 15 – 24 yrs		0.85		
Percent completing literacy test		-0.58		
Single mother families		0.90		
Percent aboriginal (log)		0.74		
Average earnings			0.80	
25+ years with only high school education			-0.41	
Value of dwellings			0.56	
Ratio of pop 65+ to pop 25 – 64 years			-1.01	
Population growth 96 – 01			0.64	
Percent of labour force in small workplaces				0.65
Percent of youth in small workplaces				0.72

* Only regression coefficients of over 0.50 are reported in the table, except for situations where a factor loading of less than 0.50 is the highest loading for a given variable.

**Table 3:** Description of factors and typical and atypical regions

**Factor**	**Description**	**Typical regions**	**Atypical regions**
One	low population density, low youth education, low spending on household expenses, high household ownership and low population mobility	1. Rainy river district 2. Bruce county 3. Kenora district 4. Manitoulin and Sudbury districts	1. Toronto division 2. Peel Region 3. Ottawa division 4. Frontenac county 5. York region
Two	High union membership, high youth unemployment, low household ownership, low literacy test completion, high single mother families, high aboriginal population	1. Kenora district 2. Cochrane district 3. Nipissing district 4. Thunder Bay district	1. York region 2. Halton region 3. Huron county 4. Dufferin county 5. Perth county
Three	High earnings, high adult education, high value of dwellings, young population, high population growth	1. York region 2. Halton region 3. Peel region 4. Ottawa division 5. Durham region	1. Timiskaming district 2. Haliburton & Parry Sound districts 3. Manitoulin & Sudbury districts 4. Algoma district
Four	High number of small workplaces, high spending on household expenses	1. Manitoulin & Sudbury districts 2. Muskoka district 3. Haliburton & Parry Sound districts 4. Dufferin county	1. Halton region 2. Durham region 3. Frontenac county 4. Essex county 5. Peel region

Table 3 describes each of the four factors, and lists typical and atypical census divisions – based on factor scores (atypical regions are those with the lowest factor scores and typical are those with the highest factor scores).

Table 4 presents the univariate and multivariate regressions for each of the factors on the adjusted claim rate. Regression diagnostics suggested multicollinearity existed between factor three and the other factors in the model (VIF = 5.05), therefore this factor was removed from the multivariate model. The strongest relationship was found between factor four and the adjusted injury rate.

**Table 4:** Unstandardized beta estimates between adjusted injury rate and all four factors.

**Variable**	**Univariate**			**Multivariate**		
	**Est**	**se**	**T-stat**	**Est**	**se**	**T-stat**

Factor One	-0.13	0.06	-2.21	-0.06	0.06	-1.02
Factor Two	-0.07	0.09	-0.79	-0.04	0.08	-0.59
Factor Three	0.18	0.07	2.54	--	--	--
Factor Four	-0.35	0.10	-3.45	-0.30	0.12	-2.58

Table 5 presents the univariate and multivariate regression estimates for the best variable within each factor. The best variable is defined as the variable within a factor that was most strongly associated with the adjusted injury rate, using manual backwards stepwise regression. In the best fitting multivariate regression model, more residential stability was associated with lower work injury rates for young people. In addition, proportionally fewer small workplaces in a region was associated with lower work injury rates. Note the univariate and multivariate regression estimates changed due to the correlations between the variables included in the regression. For example, percent of labour force in small workplaces was correlated with ratio of population aged over 65 yrs to population aged 15 – 64 yrs (r = 0.50), and with percent of population living at the same address for the last 5-years (r = 0.54). The ratio of the population aged over 65 years to the population aged 15 – 64 years was positively correlated with the percent of the population living at the same address for the last 5 years (r = 0.70). However, the regression diagnostics did not indicate substantial multicollinearity between study variables

**Table 5:** Unstandardized beta estimates for best fitting model using best individual predictors within each factor and injury rate

**Variable**	**Univariate**			**Multivariate**		
	**Est**	**se**	**T-stat**	**Est**	**se**	**T-stat**

Factor 1: Percent of population living at the same address for the last 5-years	-6.04	1.61	-3.76	-3.84	1.84	-2.09
Factor 2: Unemployment rate 15 to 24 years old	-0.05	0.03	-1.46	--	--	--
Factor 3: Ratio of the population aged over 65 years to population aged 15 to 64 years	-5.50	1.56	-3.53	--	--	--
Factor 4: Percent of labour force in small workplaces	-0.05	0.01	-3.84	-0.0318	0.014	-2.20

To examine further regional differences, we examined the distribution of types of injuries by the claim rate groupings shown in Figure 1. Table 6 shows that regions with the lowest claim rates tended to have proportionally fewer cuts and burns than higher claim rate regions.

**Table 6:** Nature of injury by regions – grouped according to claim rate grouping in Figure 1.

	**Lowest rate region**		**Lower rate region**		**Middle rate region**		**High rate region**		**Highest rate region**	
**Nature of Injury**	**N**	**%**	**N**	**%**	**N**	**%**	**N**	**%**	**N**	**%**
Cuts	45	15.1%	217	16.3%	716	19.7%	767	17.0%	750	19.1%
Burns	10	3.3%	75	5.6%	168	4.6%	221	4.9%	168	4.3%
Fractures	--	--	90	6.7%	191	5.3%	252	5.6%	209	5.3%
Amputations	--	--	13	1.0%	40	1.1%	50	1.1%	36	0.9%
Sprains & Strains	101	33.8%	409	30.7%	1089	29.9%	1396	30.9%	1214	30.9%
Contusions	39	13.0%	154	11.5%	544	15.0%	619	13.7%	521	13.2%
Other	104	34.8%	376	28.2%	890	24.5%	1208	26.8%	1035	26.3%

-- supressed due to low cell counts

## Discussion

Few studies to date have examined geographic variation in occupational health outcomes. The substantial geographic variation in workers' compensation claims among Ontario young workers is consistent with the previous research on young workers in Massachusetts [36]. Further, the results of the present study showed substantial geographic variation in youth work injury rates even after controlling for several demographic and job characteristics. This residual variation raises the possibility that particular contextual factors associated with the regions influence youth work injury rates above and beyond the compositional differences in jobs and youth between regions.

This study contributes to the literature by also investigating the relationship between regional ecological features and youth work injury. Unlike the previous study of Massachusetts that found that certain indicators of area deprivation such as unemployment or low education level were related to elevated work injury rates [36], we found other area indicators to be salient. Of course cross-jurisdictional comparison of young worker studies is difficult, which is partially due to jurisdictional differences in the definition and reporting practices for a lost-time claim. Also specifically for teenage workers, jurisdictions have different minimum age regulations that influence where youth can work. For example, the US. Fair Labour Standards Act imposes restrictions on the types of equipment and tasks youth can perform, and some states have more stringent regulations. In Canada, primary responsibility for occupational heath and safety falls on the provinces. Regulations in Ontario are framed in terms of the minimum age to work in certain industries. For instance, 14 year olds can work in offices and stores, but one cannot work in construction settings until 16 years of age. In the present study, the minimum age regulations were uniform across Ontario, so only differences in compliance could contribute to the regional variation observed.

The hypotheses about area deprivation and work injury were partially supported. For example greater residential stability in an area was associated with lower youth work injury rates, controlling for other regional socioeconomic factors and industrial/occupational mix. A possible explanation for this association is that greater residential stability is also linked to longer job tenures in the area, with greater job experience being correlated with lower injury rates [53]. Other potential factors include better workplace safety climates because of greater social cohesion in the area.

We found that census divisions with a greater proportion of small workplaces (defined workplaces with less than 20 employees) had lower youth work injury. However, this finding should not be taken as contradictory evidence that smaller workplaces have higher injury rates than larger workplaces, which has been found when comparing workplace injury rates [54,55]. First, our analysis is at the ecological level, and as such relationships found at the group level should not be extended to the level of the individual (i.e. the ecological fallacy). Second, a closer look at the regions that had a greater proportion of small workplaces was that they also tended to be some of the more affluent regions in Ontario (e.g., Muskoka) and tended to have greater residential stability as well. Further, there may be other area-level factors, which we are unable to include in our model, which are also associated with lower injury rates and higher proportion of smaller workplaces.

There was a differential distribution of types of injury, with low-injury regions showing somewhat fewer cuts and burns than high-injury regions. This pattern may indicate that different work processes or more automated equipment may be used in certain regions. It also suggests the specific injury mechanisms prevention efforts should be targeted in high-injury regions.

One strength of this study is identifying a useful level of aggregation of youth work injuries. Contextual factors influencing work injury rates across many workplaces are difficult to observe at a small spatial scale such as the neighborhood [56]. Further, institutions such as workers' compensation boards and labour inspectorates have the ability to address different regions according to their needs.

Nevertheless, these results need to be considered in light of several methodological limitations. The use of regional breakdowns of lost-time claims from youth assumes no systematic differences in claim reporting practices by region. It also assumes that youth live and work in the same region, which appears to be the case [57]. Even though some studies have found the work injury rates and risk factors of adolescent and young adults comparable [34], future research may consider exploring differences between the two age groups. Also, while FTEs as a denominator are a useful measure of on-the-job exposure for worker subgroups where part-time work is common, there may be other factors associated with part-time work that make hours worked part-time qualitatively different than full-time work. Finally, larger and populous regions may have greater heterogeneity than other regions, which suggests that studies with individual level data that allow multi-level analyses would be useful. Nevertheless this ecological study provides preliminary information on factors to explore in future research and this method is justified given the dearth of research on this topic.

## Conclusion

In sum, descriptive findings showed substantial geographic variation in young worker injury rates, even after controlling for several job and demographic variables. Region-level characteristics such as greater residential stability were associated with low work injury rates. From a policy perspective, the non-uniform regional distribution of youth work injuries in a jurisdiction can aid prevention resource allocation. The association between area deprivation and youth work injury raises two possible implications. First, it suggests that work injury prevention strategies can be integrated with other local economic development measures. Second, it suggests that relevant authorities might examine whether work safety measures are unevenly distributed with respect to their socio-economic environments as well.

The impact of prevention activities also need to consider both the relative work injury rate of a region and the population size. For example, small reductions in the work injury rate of a populous region with a mid-range rate may lead to greater absolute number of claims prevented than reducing the risk in regions with the highest work injury rates, if they are of low population density.

It would be worthwhile for future research to examine trends in geographic variation, which would help identify which ecological correlates covary over time with work injury rates. In addition, examining the regional distribution of adult work injury rates would aid in determining how generic or unique the ecological correlates of young worker injuries are.

## Abbreviations

CD – census division

FTE – Full-time equivalent

SLID – Survey of Labour and Income Dynamics

OSSLT – Ontario Secondary School Literacy Test

WSIB – Workplace Safety and Insurance Board

CI – 95% confidence intervals

## Competing interests

The author(s) declare that they have no competing interests.

## Authors' contributions

FCB conceived of the study and drafted parts of the manuscript. PS acquired the data, carried out the analyses, and drafted parts of the manuscript. JD contributed to the analysis/interpretation of the data and revised the manuscript for important intellectual content. All authors have read and approved the final manuscript.

## Pre-publication history

The pre-publication history for this paper can be accessed here:



## Supplementary Material

Additional file 1 Appendix A. Lost time claim rates by Census division. This table provides the lost-time claim rates, claim counts and confidence intervals by census division. Click here for file

Additional file 2 Appendix B. Map of census divisions in Ontario. This map shows the census divisions in Ontario, with a legend below to identify by name the regions colour-coded in Figure 1 Click here for file

## References

[B1] Diez-Roux AV, Merkin S, Arnett D, Chambless L, Massing M, Nieot F, Sorlie P, Szklo M, Tyroler HA, Watson RL (2001). Neighborhood of residence and incidence of coronary heart disease. N Eng J Med.

[B2] Macintyre S, Ellaway A, Kawachi I, Berkman L (2003). Neighborhoods and health: an overview. Neighborhoods and health.

[B3] Dunn J, Frohlich K, Ross N, Curtis L, Sanmartin C, Heymann J, Hertzman C, Barer M, Evans R (2005). Role of geography in inequalities in health and human development. Healthier Societies: From Analysis to Action.

[B4] Keating DP, Hertzman C (1999). Developmental health and the wealth of nations: social, biological and educational dynamics.

[B5] Leventhal T, Brooks-Gunn J (2000). The neighborhoods they live in: the effects of neighborhood residence on child and adolescent outcomes. Psychol Bull.

[B6] Duncan G, Raudenbush S (1999). Assessing the effects of context in studies of child and youth development. Educ Psych.

[B7] Brooks-Gunn J, Duncan G, Klebanov P, Sealand N (1993). Do neighbourhoods influence child and adolescent development. Am J Sociol.

[B8] Pickett KE, Pearl M (2001). Multilevel analyses of neighbourhood socioeconomic context and health outcomes: a critical review. J Epidemiol Community Health.

[B9] Jones K, Duncan C (1995). Individuals and their ecologies: Analysing the geography of chronic illness within a multilevel modelling framework. Health & Place.

[B10] Durkheim E (1938). The Rules of Sociological Method.

[B11] Durkheim E, Spaulding JA, Simpson G (1951). Suicide: A Study in Sociology.

[B12] Rose G (1985). Sick individuals and sick populations. Int J Epidemiol.

[B13] Rose G (1992). The strategy of preventive medicine.

[B14] Schwartz S, Diez-Roux AV (2001). Commentary: Causes of incidence and causes of cases-a Durkheimian perspective on Rose. Int J Epidemiol.

[B15] Schwartz S (1994). The fallacy of the ecological fallacy: the potential misuse of a concept and the consequences. AJPH.

[B16] Usalcas J Youth and the labour market. Perspectives 2005 75 001 XIE.

[B17] Statistics Canada (2001). Canadian Labour Force Survey, public use files. Catalogue No. 71M0001XCB.

[B18] Bowlby G (2000). The school-to-work transition. Catalogue no 75-001-XPE.

[B19] Laflamme L, Menckel E (1995). Aging and occupational accidents: a review of the literature of the last three decades. Saf Sci.

[B20] Salminen S (2004). Have young workers more injuries than older ones? An international literature review. J Saf Res.

[B21] Breslin FC, Smith P (2005). Age-related differences in work injuries: A multivariate, population-based study. Am J Ind Med.

[B22] Castillo D, Landen D, Layne LA (1994). Occupational injury deaths of 16- and 17-year-olds in the United States. Am J Pub Health.

[B23] Ruser J (1998). Denominator choice in the calculation of workplace fatality rates. Am J Ind Med.

[B24] Parker DL, Carl WR, French LR, Martin FB (1994). Characteristics of adolescent work injuries reported to the Minnesota Department of Labor and Industry. Am J Pub Health.

[B25] Parker DL, Carl WR, French LR, Martin FB (1994). Nature and incidence of self-reported adolescent work injury in Minnesota. Am J Ind Med.

[B26] Breslin F, Tompa E, Zhao R, Amick B, Pole J, Smith P, Hogg-Johnson S Work disability absence among young workers leads to earnings losses in the following year. Scand J Work Environ Health.

[B27] Belville R, Pollack SH, Godbold J, Landrigan PJ (1993). Occupational injuries among working adolescents in New York State. JAMA.

[B28] Schober SE, Handke JL, Halperin WE, Moll MB, Thun MJ (1988). Work-related injuries in minors. Am J Ind Med.

[B29] Banco L, Lapidus G, Braddock M (1992). Work-related injury among Connecticut minors. Pediatrics.

[B30] Horwitz IB, McCall BP (2005). Occupational injury among Rhode Island adolescents: an analysis of workers' compensation claims, 1998 to 2002. J Occup Environ Med.

[B31] Brooks DR, Davis LK (1996). Work-related injuries to Massachusetts teens, 1987–1990. Am J Ind Med.

[B32] Miller ME, Kaufman JD (1998). Occupational injuries among adolescents in Washington State, 1988–1991. Am J Ind Med.

[B33] Simoyi P, Islam S, Haque A, Meyer J, Doyle E, Ducatman A (1998). Evaluation of occupational injuries among young workers in West Virginia. Hum Ecol Risk Assess.

[B34] Breslin F, Koehoorn M, Smith P, Manno M (2003). Age-related differences in work injuries and permanent impairment: A comparison of workers' compensation claims among adolescents, young adults, and adults. Occup Environ Med.

[B35] Breslin F, Smith P, Mustard C, Zhao S (2006). Young people and work injuries: an examination of jurisdictional variation within Canada. Inj Prev.

[B36] Brooks DR, Davis LK (1996). Work-related injuries to Massachusetts teens, 1987–1990. Am J Ind Med.

[B37] Statistics Canada (1992). Standard geographical classification 1991 – the classification.

[B38] Castree N, Coe N, Ward K, Samers M (2006). Spaces of Work: Global Capitalism and Geographies of Labour.

[B39] Muntaner C, Li Y, Xue X, Thompson T, O'Campo P, Chung H, Eaton WW (2006). County level socioeconomic position, work organization and depression disorder: A repeated measures cross-classified multilevel analysis of low-income nursing home workers. Health & Place.

[B40] Boyle MH, Georgiades K, Racine Y, Mustard C (2007). Working paper #306 Neighborhood and family influences on educational attainment: results from Ontario Child Health Study, follow-up 2001.

[B41] Gordon D (1995). Census based deprivation indices: Their weighting and validation. J Epidemiol Comm Health.

[B42] Pampalon R, Raymond G (2000). A deprivation index for health and welfare planning in Quebec. Chron Dis Can.

[B43] Eakin JM, Lamm F, Limborg HJ, Frick K, Jensen PL, Quinlan M, Wilthagen T. Pergamon (2000). International perspective on the promotion of health and safety in small workplaces. Systematic occupational health and safety management Perspectives on an international development.

[B44] Vector Research Workers Health and Safety Centre (2000). Opinion poll on workplace health and safety conditions in Ontario.

[B45] Statistics Canada (2000). A guide to the Labour Force Survey. http://www.statcan.ca/english/IPS/Data/71-543-GIE.htm.

[B46] Jackson LL (2001). Non-fatal occupational injuries and illnesses treated in hospital emergency departments in the United States. Inj Prev.

[B47] Sullivan T, Sinclair S, Allingham R (2001). Health, safety and injuries in Canada. Presentation at the Melbourne Invitational Seminar, Syndey Australia.

[B48] Smith P, Mustard C, Payne J (2004). Methods for estimating the labour force insured by the Ontario Workplace Safety & Insurance Board: 1999–2000. Chron Dis Can.

[B49] Herbert F, Duguay P, Massicotte P, Levy M (1996). Révision des catégories professionnelles utilisées dans les études de I'IRSST portant sur les indicateurs quinquennaux de lésions professionnelles. Quebe, IRSST.

[B50] Hennekens CH, Buring JE (1987). Epidemiology in medicine.

[B51] Statistics Canada (1989). Canadian Work Injuries Standard.

[B52] Statistics Canada (1997). Survey of Labour and Income Dynamics: Microdata user's guide.

[B53] Breslin C, Smith P (2006). Trial by fire: a multivariate examination of the relation between job tenure and work injuries. Occup Environ Med.

[B54] Shannon HS, Vidmar M (2004). How low can they go? Potential for reduction in work injury rates. Inj Prev.

[B55] Salminen S, Saari J, Saarela KL, Rasanen T (1993). Organizational factors influencing serious occupational accidents. Scand J Work Environ Health.

[B56] Graham D, Glaister S, Anderson R (2005). The effects of area deprivation on the incidence of child and adult pedestrian casualties in England. Acc Analysis Prev.

[B57] Sullivan M (1989). Getting paid: youth crime and work in the inner city.

