# Supplementary material for: An ecological study of regional variation in work injuries among young workers
Source: BMC Public Health. 2007 May 23;7:91. doi: 10.1186/1471-2458-7-91 (PMC1894966; doi:10.1186/1471-2458-7-91)
Supplement: Additional file 1 — Appendix A. Lost time claim rates by Census division. This table provides the lost-time claim rates, claim counts and confidence intervals by census division. [file 1471-2458-7-91-S1.docx]

Appendix A
