# Supplementary material for: An ecological study of regional variation in work injuries among young workers
Source: BMC Public Health. 2007 May 23;7:91. doi: 10.1186/1471-2458-7-91 (PMC1894966; doi:10.1186/1471-2458-7-91)
Supplement: Additional file 2 — Appendix B. Map of census divisions in Ontario. This map shows the census divisions in Ontario, with a legend below to identify by name the regions colour-coded in Figure 1 [file 1471-2458-7-91-S2.docx]

Appendix B – Map of census divisions in Ontario


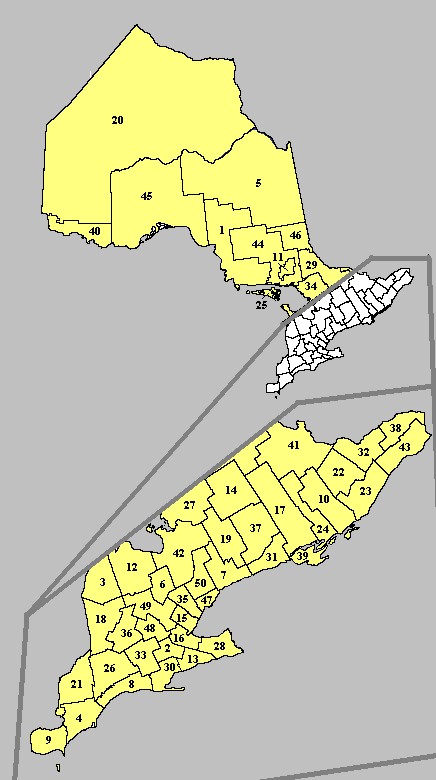


Appendix B – Legend for map of census divisions in Ontario

1. Algoma (pop. 118,567)
2. Brant (pop. 118,485)
3. Bruce (pop. 63,892)
4. Chatham-Kent (pop. 107,709)
5. Cochrane (pop. 85,247)
6. Dufferin (pop. 51,013)
7. Durham (pop. 506,901)
8. Elgin (pop. 81,553)
9. Essex (pop. 374,975)
10. Frontenac (pop. 138,606)
11. Greater Sudbury (pop. 155,268)
12. Grey (pop. 89,073)
13. Haldimand (pop. 43,823)
14. Haliburton (pop. 15,085)
15. Halton (pop. 375,229)
16. Hamilton (pop. 490,268)
17. Hastings (pop. 125,915)
18. Huron (pop. 59,701)
19. Kawartha Lakes (pop. 69,179)
20. Kenora (pop. 61,802)
21. Lambton (pop. 126,971)
22. Lanark (pop. 62,495)
23. Leeds and Grenville (pop. 96,606)
24. Lennox and Addington (pop. 39,461)
25. Manitoulin (pop. 12,679)
26. Middlesex (pop. 403,185)
27. Muskoka (pop. 53,106)
28. Niagara (pop. 410,574)
29. Nipissing (pop. 82,910)
30. Norfolk (pop. 60,847)
31. Northumberland (pop. 77,497)
32. Ottawa (pop. 774,072)
33. Oxford (pop. 99,270)
34. Parry Sound (pop. 39,665)
35. Peel (pop. 988,948)
36. Perth (pop. 73,675)
37. Peterborough (pop. 125,856)
38. Prescott and Russell (pop. 76,446)
39. Prince Edward (pop. 24,901)
40. Rainy River (pop. 22,109)
41. Renfrew (pop. 95,138)
42. Simcoe (pop. 377,050)
43. Stormont, Dundas and Glengary (pop. 109,522)
44. Sudbury (pop. 22,894)
45. Thunder Bay (pop. 150,860)
46. Timiskaming (pop. 34,442)
47. Toronto (pop. 2,481,494)
48. Waterloo (pop. 438,515)
49. Wellington (pop. 187,313)
50. York (pop. 729,254)
